# Pakistan’s unsafe medical injections and the enduring public health crisis

**DOI:** 10.12669/pjms.41.12.13388

**Published:** 2025-12

**Authors:** Arshad Altaf, Sharaf Ali Shah

**Affiliations:** 1 Arshad Altaf, WHO Regional Office for the Eastern Mediterranean, Cairo, Egypt; 2 Sharaf Ali Shah, Bridge Consultants Foundation, Karachi, Pakistan

## SUMMARY

- Unsafe medical injections in Pakistan transmit infections.
- The proposed solutions are not complex to implement.
- They include rational practices, longer outpatient hours of government health centers and empowering the community with knowledge.

## INTRODUCTION

A medical injection is generally considered a source of relief from an acute or chronic illness or a disease. The World Health Organization (WHO) defines a safe injection as the one that does not harm the recipient, does not expose the provider to risk and does not result in waste that is dangerous to others.^1^ However, unsafe medical or therapeutic injections given by healthcare providers are a harsh reality and a common practice in South Asia.^2^ One way that an injection can be unsafe is the reuse of the same syringe on more than one patient to save money. This is usually done by private healthcare providers who charge a fee to the patients. The fee sometimes takes the form of a package that includes the provider’s fee, syrup and tablets, and an injection, which could be intramuscular or intravenous. Often, the patients are unaware whether the syringe or the needle that is used on them has been used on another patient.^3^ This has the risk of transmitting bacterial infections, such as an abscess on the injection site and more serious consequences include transmission of hepatitis B, C and human immunodeficiency virus (HIV) infections.^4^ Incorrect injection technique can also cause nerve damage. This article comments on unsafe medical injection practices in Pakistan and their role in spreading infection.

### Injections spreading infections in Pakistan:

Unsafe medical injections worldwide have improved. Hayashi and colleagues reviewed the results of Demographic and Health Surveys (DHS) of 40 of 92 countries that reported on injection practices from 2011 to 2015. The survey respondents reported 1.64 injections per year. Of these, 96.1% were reported to be given with devices taken out of new packaging. The Eastern Mediterranean Region (EMR) of the WHO had the highest average number (3.8 injections) per year compared to 2.6 for South-East Asia or 1.6 for the Western Pacific, 1.1 for Africa and 1.2 for the Americas regions. The analysis revealed that since 2011, the number of unsafe medical injections in Pakistan has not changed significantly.^5^ This is noteworthy because Pakistan has the second-highest burden of Hepatitis-C infections in the world.^6^ The average number of injections per person per year in Pakistan has been estimated between 8-13.8.^7^,^8^

WHO’s Global Hepatitis Report 2024 estimated the contribution of unsafe medical injections to Hepatitis-C transmission in 60 countries, representing 45% of the world’s population. Pakistan accounted for 44% of all new Hepatitis-C infections attributed to unsafe medical injections in these 60 countries.^9^

In April 2019, in rural Sindh province of Pakistan, an HIV outbreak impacted the lives of children and women. A multi-agency team of public health specialists and clinicians investigated and reported that by the end of 2019, out of 21,962 screened children, 881 (4.0%) were tested positive for HIV.^10^ Investigation into the factors associated revealed that parenteral route, probably because of unsafe injection practices by private practitioners as the predominant mode of HIV transmission.^10^ A systematic review by Rabold and colleagues analyzed reported HIV outbreaks in Pakistan from 2000 to 2019.^11^ They reported iatrogenic transmission from unsafe injection practices as the main contributing factor in five of the seven outbreaks.^11^ In 2025, in the Taunsa district of Punjab province, 150 children between six months and 10 years were tested HIV positive in a three-month period. The children had no family history of HIV and the most common identified risk factor was exposure to medical treatment and vaccination history. Further investigation pointed towards exposure to unsafe injection practices.^12^ Studies have documented that patients in Pakistan like to receive injections.^13^,^14^ At the same time, healthcare providers prefer to prescribe injections to provide quick relief. For a private practitioner, an injection in a prescription generates extra income.^14^,^15^ Patients visiting untrained private healthcare providers were more likely to receive injections than those visiting providers in the public sector.^15^

## SOLUTIONS

### Community empowerment:

Curtailing the medical practices of untrained healthcare providers has been a challenge in the presence of a lax regulatory system. Community empowerment is of value because the social structure in societies like Pakistan is such that the patient dare not question the healthcare provider and is often intimidated from questioning anything that is coming from the person holding or sporting a stethoscope around the neck. Community empowerment has shown some positive results. If the patient is empowered with knowledge, can break the social power barrier, and can question the provider about the need for an injection and the type of syringe used, it can help reduce the number of unnecessary injections and the reuse of injection equipment. A multi-pronged approach in rural Pakistan improved misconceptions about infection transmission and positively showed that the community’s perspective about a new syringe changed.^16^

### Enforcing rational practices:

Untrained healthcare providers include the person pretending to be a doctor and often have a helper who should ideally be a trained paramedic, but is not and literally learns how to provide injections and other minor medical procedures on the job. A quick search will reveal numerous trainings on infection prevention and control across the country that are organized for them, but the impact is not visible. As is evident from the most recent HIV outbreak reported earlier.

Anyone pretending to be a trained doctor and without a medical qualification should not be allowed to examine patients and prescribe medicines and injections.

### Outpatient hours of primary and secondary level facilities:

Across Pakistan, in the rural and peri-urban settings, government primary healthcare centers close between 1:00 and 2:00 pm, sometimes even earlier. This has led to the mushroom growth of private practitioners, both trained and untrained. These health facilities have to remain open till late in the afternoon to provide services for a longer duration.

### WHO recommendation:

The 2016 WHO guideline on injection safety recommends the use of syringes with a re-use prevention feature (RUP devices) to mitigate the risk of re-use.^17^ However, this would require an active engagement with the injection provider, another daunting task in a lax regulatory system.

## CONCLUSION

The problem of unsafe injections and transmission of hepatitis B and C in Pakistan has been reported as far back as 2000.^18^ A quarter of a century later, the problem still exists and affects patients and communities. None of the proposed solutions is difficult to implement and enforce. Countless lives can be saved if this problem is addressed sooner rather than later.
